# Sarcopenia in Patients with Rheumatic Diseases: Prevalence and Associated Risk Factors

**DOI:** 10.3390/jcm7120504

**Published:** 2018-12-01

**Authors:** Michele Barone, Maria Teresa Viggiani, Maria Grazia Anelli, Rosalinda Fanizzi, Orsola Lorusso, Giuseppe Lopalco, Luca Cantarini, Alfredo Di Leo, Giovanni Lapadula, Florenzo Iannone

**Affiliations:** 1 Gastroenterology Unit, Department of Emergency and Organ Transplantation, University of Bari, 70124 Bari, Italy; viggiani.mt@libero.it (M.T.V.); orsolalorusso@me.com (O.L.); alfredo.dileo@uniba.it (A.D.L.); 2 Rheumatology Unit, Department of Emergency and Organ Transplantation, University of Bari, 70124 Bari, Italy; marynelli@libero.it (M.G.A.); rosalinda.fanizzi@hotmail.it (R.F.); glopalco@hotmail.it (G.Lo.); giovanni.lapadula@uniba.it (G.La.); florenzo.iannone@uniba.it (F.I.); 3 Research Center of Systemic Autoinflammatory Diseases and Behḉet’s Disease Clinic, Department of Medical Sciences, Surgery and Neurosciences, University of Siena, 53100 Siena, Italy; cantariniluca@hotmail.com

**Keywords:** rheumatoid arthritis, psoriatic arthritis, ankylosing spondylitis, muscle mass, muscle strength, physical disability, biologic therapy

## Abstract

The prevalence of sarcopenia in rheumatic diseases has been evaluated in single diseases using various diagnostic approaches, generating conflicting data on the pathogenetic mechanism(s). Herein, we evaluated both muscle mass index (MMI) and muscle strength to assess sarcopenia and presarcopenia in patients with rheumatoid arthritis (RA), psoriatic arthritis (PsA), and ankylosing spondylitis (AS). Moreover, we evaluated the possible impact of disease/patient-related characteristics, therapeutic regimens, and nutritional aspects on sarcopenia. The present study included 168 patients of both genders, aged 40–75 years. All patients underwent a nutritional evaluation, physical activity level assessment, rheumatologic evaluation, and an MMI and muscle strength assessment. The prevalence of sarcopenia was about 20% in all the three rheumatologic diseases, whereas presarcopenia was significantly different in RA, PsA and AS (*p* = 0.006). At multivariate analysis, only age ≥60 years and the presence of a disability were associated with a significantly increased risk of sarcopenia (*p* = 0.006 and *p* = 0.01, respectively), while a higher C-reactive protein did not reach statistical significance. Sarcopenia is similar in RA, PsA and AS, whereas presarcopenia significantly differs in these three diseases. Disease activity/inflammation and nutritional aspects do not influence sarcopenia, while age ≥60 years and the presence of a disability significantly increase the risk of sarcopenia.

## 1. Introduction

The concept of sarcopenia was first developed to describe the age-related decrease in muscle mass in elderly human subjects (“primary sarcopenia”). Subsequently, sarcopenia has been studied in several clinical settings (usually referred to as “secondary sarcopenia”), recognizing three main pathogenic mechanisms: inflammatory activity, nutritional deficit, and physical activity impairment [1]. However, although there is a widely accepted definition of primary sarcopenia, an agreement in research and clinical practice is still lacking for secondary sarcopenia [1,2]. Currently, sarcopenia is defined by European working group on sarcopenia in older people (EWGSOP) criteria as an abnormally low muscle mass associated with low skeletal muscle strength and/or low physical performance, leading to an increased risk of physical disability, poor quality of life, and death [1]. Notably, recent evidence suggests that the correlation between “muscle quantity” (muscle mass) and “muscle function” (muscle strength or physical performance) is relatively weak, and, in contrast to muscle function, muscle mass has been demonstrated to be a poor predictor of functional limitation, gait speed, and also mortality [3]. 

In rheumatic diseases, several studies have measured the muscle mass index (MMI), without taking into consideration the assessment of muscle strength and/or physical performance [4,5,6,7,8,9,10]. On the other hand, three more recent studies used EWGSOP criteria for the assessment of sarcopenia [11,12,13]. However, the latter studies were focused on three different rheumatic diseases and used different techniques for the assessment of muscle mass (Dual energy X-ray absorptiometry (DXA) or bioelectrical impedance analysis (BIA)) and muscle function (handgrip and/or one physical performance test) [11,12,13], making it difficult to compare them. In addition, the data about the causes of sarcopenia and the possible beneficial effect of therapies with antibodies targeting specific immune system pathways (biological therapy) are not conclusive [11,13].

In the present study, we assessed sarcopenia and presarcopenia on the basis of EWGSOP criteria and compared, for the first time, these two conditions in three different rheumatic diseases: rheumatoid arthritis (RA), ankylosing spondylitis (AS) and psoriatic arthritis (PsA) (primary end-point). In addition, we evaluated the influence of age, sex, physical activity, nutritional intake, disease duration, disease activity, disability, and type of therapy on sarcopenia (secondary end-point).

The comparison of sarcopenia and presarcopenia in patients affected by RA, PsA, and AS led to the demonstration that the prevalence of sarcopenia is similar in the three diseases, while presarcopenia is significantly increased in PsA and AS when compared to RA. Our analysis suggests that age and disability are the only significant risk factors for sarcopenia.

## 2. Patients and Methods 

### 2.1. Patients Characteristics

Caucasian rheumatologic patients included in a prospective register (ClinicalTrials.gov identifier NCT01543594) underwent a nutritional evaluation by the nutritional team of the Gastroenterology Unit from May 2015 to December 2016. Our research was carried out in compliance with the Helsinki Declaration, all patients gave their informed consent, and all procedures received the local ethics committee’s approval (protocol# 589CE).

Patients aged 40–75 years of both sexes with a diagnosis of RA, SA, and PsA were enrolled in order to evaluate the prevalence of sarcopenia and presarcopenia in these pathological conditions. The exclusion criteria were: uncertain diagnosis; obesity (the assessment of sarcopenia by anthropometry has not been validated in the presence of this condition) [14]; possible other causes of secondary sarcopenia (malnutrition, decompensated diabetes, advanced chronic kidney disease, chronic obstructive pulmonary disease, and chronic heart failure); presence of severe metacarpophalangeal joint derangement (it would be a major confounder of grip strength).

During the nutritional visit, weight, height, body mass index, (BMI), total calories and protein intake, physical activity level (PAL), MMI and muscle strength were evaluated. The rheumatologic visit involved the evaluation of disability and disease activity scores, and erythrocyte sedimentation rate (ESR) and C-reactive protein (CRP) determination. The data of diagnosis, duration of illness, current rheumatic therapy, and presence/absence of rheumatoid factor (RF) and anti-citrulline antibodies (anti-CCP Ab) were obtained from outpatient medical records.

### 2.2. Nutritional Evaluations

In addition to weight, height, and BMI, a quantitative and qualitative evaluation of the dietary intake was performed as previously described, and the daily calorie and protein intake were adjusted for body weight [15]. PAL was obtained by calculating the ratio of total to basal daily energy expenditure (TEE/BEE). BEE was calculated using the Harris–Benedict formula and TEE was calculated using BEE and the integrated energy index (IEI). The data on the activity and occupation of all participants of the study were gathered for the entire 24 h to determine the IEI [16]. The measurement of MMI was based on the determination of body composition assessed by bioelectrical impedance analysis (BIA) (BIA 101, Akern srl, Pontassieve, (FI), Italy) as previously described [17]. This technique correlates well with dual-energy X-ray absorptiometry (DXA) in this clinical setting [7]. In addition, MMI was evaluated by anthropometry. The total muscle mass evaluation by anthropometry was calculated as previously described by Lee et al. [14]. MMI was obtained as the ratio of total muscle mass to height^2^. A handgrip dynamometer (Jamar®, Sammons Preston, Bolingbrook, IL, USA) was used to determine muscle strength, as previously described [17]. The test was repeated three consecutive times, for each forearm, and the mean value was used.

### 2.3. Sarcopenia Evaluation

The diagnosis of sarcopenia was performed in the presence of both muscle mass and muscle strength reduction, while muscle mass reduction alone without muscle strength impairment was considered presarcopenia [1,18]. The reference values for the diagnosis of muscle mass and muscle strength reduction were: MMI < 6.75 kg/m^2^ in women and <10.75 kg/m^2^ in men (associated with physical disability in subjects aged ≥60 years) and handgrip strength <20 kg in women and <30 kg in men (associated to reduced mobility), respectively [19,20].

### 2.4. Disease Activity Assessment

The disease activity was evaluated using the following scores: “disease activity score 28 (DAS 28)” [21,22], “clinical disease activity index (CDAI)”, and “simplified disease activity index (SDAI)” in RA patients [23,24,25]; “Bath Ankylosing Spondylitis Disease Activity Index (BASDAI)” [26], “Ankylosing Spondylitis Disease Activity Score-CRP (ASDAS-CRP)”, and “Ankylosing Spondylitis Disease Activity Score-ESR (ASDAS-ESR)” for AS patients [27,28]; “Disease Activity Index for Psoriatic Arthritis (DAPSA)”, for PsA patients [29,30]. For the assessment of disability, the “Health assessment questionnaire (HAQ)” [31,32] was used. CRP was considered pathological when the ratio between the value found in each subject and the upper normal limit was >1.

### 2.5. Statistical Analysis

For continuous variables, an analysis of sample distribution was performed by evaluating the symmetry with the Skewness and Kurtosis tests, and the variables were expressed as mean ± standard deviations (SD). Statistical comparisons of two or more continuous variables with normal distribution were performed by t test and ANOVA, respectively. Categorical variables were expressed as percentages and compared using the chi-square or Fisher’s exact test, followed by the Bonferroni post-hoc analysis when required. The evaluation of factors associated with sarcopenia (covariates) was performed using univariate analysis, and only covariates showing a *p* value < 0.25 at univariate analysis were included in the multivariate logistic regression analysis. The correlation between MMI values obtained with BIA and anthropometry was performed by Pearson’s correlation test. The statistical significance was set at *p* < 0.05. The analyses were performed using SPSS software, version 23.0 (SPSS Inc., Chicago, IL, USA).

## 3. Results

A total of 254 subjects were examined for eligibility, and only 168 adults were enrolled (36 were excluded due to exclusion criteria or missing data). 

In Table 1 we reported a comparison among demographic and clinical and therapeutic data in patients with RA, PA, and AS. 

In RA patients there was a higher prevalence of women, a lower level of physical activity, and a higher inflammatory state compared to PA and AS patients. Age, BMI, caloric/protein intake, disease duration, type of therapy and disability did not differ in the three rheumatic diseases. 

Among all the patients, 124 were on treatment with biologic drugs (for 47.5 ± 42.1 months) and 44 with conventional Disease Modifying Anti-Rheumatic Drugs (cDMARDS). Among the patients receiving biologic therapy, 37.0% were taking etanercept, 33.8% adalimumab and 29.0% other biologic therapy (certolizumab, ustekinumab, tocilizumab, abatacept e golimumab). In addition, in 61.3% of the cases, biologic therapy was combined with cDMARDs. The patients receiving only cDMARDs had been under therapy for 31.1 ± 32.8 months and in most of the cases were also receiving methotrexate (79.5%).

In the 76 patients with RA, the disease activity was assessed by CDAI, SDAI and DAS28 scores, resulting in remission in 43.4%, 44.7% and 47.3% of the cases, respectively. In the 70 patients with PsA, 36.7% of cases were categorized as in remission phase using the DAPSA score. In addition, in the 22 patients with AS, the use of BASDAI, ASDAS-CRP, and ASDAS-ESR detected a remission phase in 59.1%, 50.0% and 28.5% of the cases, respectively. Limiting the disease activity evaluation to the 19 patients with axial spondylitis, ASDAS-CRP and ASDAS-ESR recognized a remission phase in 42.1% and 38.8% of the cases, respectively. 

### 3.1. Prevalence of Sarcopenia and Presarcopenia

At the beginning of the study, the MMI was evaluated by both anthropometry and BIA. However, when we performed an ad interim analysis in 103 patients, the correlation between the two techniques was moderate/optimal (*r* = 0.73 by Pearson’s correlation). Therefore, we continued the study evaluating the patients’ MMI only by BIA. 

**Figure 1:**
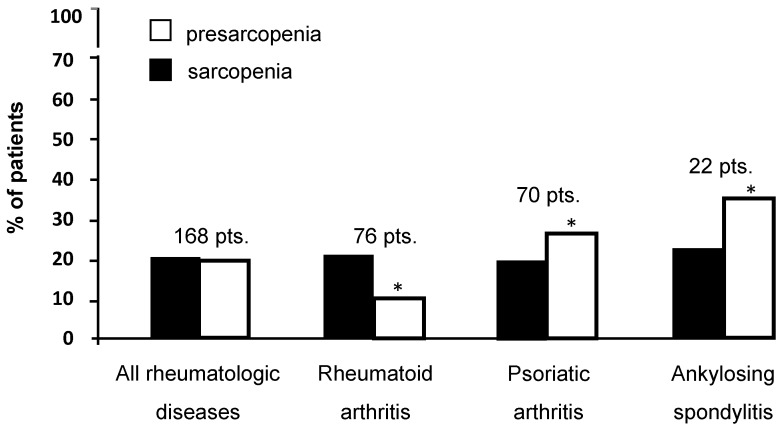
Prevalence of sarcopenia and pre-sarcopenia in all 168 patients. Pts. = patients. * *p* = 0.006 by chi-square test; RA ≠ PsA ≠ AS by Bonferroni post-hoc analysis.

As shown in Figure 1, the prevalence of sarcopenia and presarcopenia in the whole cohort of patients was 20.8% and 20.2%, respectively. However, when we analysed each disease separately (Figure 1), the prevalence of sarcopenia remained unchanged, while the prevalence of presarcopenia became significantly different among the three groups (10.5% RA, 25.7% PsA and 36.3% AS; *p* = 0.006 by chi-square test; RA ≠ PsA ≠ AS by Bonferroni post-hoc analysis). 

### 3.2. Factors Associated with Sarcopenia in Rheumatologic Diseases

At univariate analysis, the risk of sarcopenia increased in patients aged ≥60 years compared to those aged <60 years (OR = 4.3; 95% CI: 2.0–9.6, *p* < 0.001). An increased risk of sarcopenia was also observed in patients with higher CRP and in patients with disabilities (OR = 3.2; 95% CI: 1.2–8.3, *p* = 0.01 and OR = 4.5; 95% CI: 1.8–11.2, *p* = 0.001, respectively). The type of rheumatic disease, gender, calorie and protein intake, PAL, biological treatment, ESR and the duration of disease were not associated with an increased risk of sarcopenia. 

At multivariate analysis (Table 2), only age ≥60 years and the presence of a disability were associated with an increased risk of sarcopenia (OR = 3.3; CI 1.4–7.7, *p* = 0.005; OR = 3.0; 95% CI 1.2–7.9, *p* = 0.01, respectively), while a higher CRP value almost reached statistical significance (*p* = 0.07).

Surprisingly, at the univariate analysis, the patients undergoing biologic therapy had a higher percentage of sarcopenia without reaching statistical significance. Therefore, we compared patients with and without biologic therapy on the basis of the risk factors for sarcopenia (age, disability). Our results demonstrated that these patients have a higher prevalence of disability, which is an expression of a more severe/advanced disease (*p* = 0.04, by chi-square, *r*_phi_ = −0.15; *r*^2^ = 0.02). On the other hand, there was no difference concerning age.

We also evaluated the prevalence of sarcopenia in all rheumatologic patients on the basis of the different disease activity scores. When comparing patients with disease activity to patients in remission, we found no difference between the two groups, using all appropriate scores. Similarly, RF and/or anti-CCP Ab were not associated with sarcopenia in RA. On the other hand, in AS, using the BASDAI, all the 12 patients in remission did not show sarcopenia, while the remaining 10 patients with active disease were 50% sarcopenic and 50% non-sarcopenic (*p* = 0.001, by chi-square, *r*_phi_ = 0.55; *r*^2^ = 0.30). This association was not found when ASDAS-CRP and ASDAS-ESR were used. 

## 4. Discussion

To our knowledge, the evaluation of muscle mass reduction in rheumatic diseases has been previously assessed using three different approaches: (1) the comparison of MMI in patients via age- and sex-matched controls [4,6,7,10]; (2) the use of an MMI cut-off adopted to identify the primary sarcopenia [9,11,12,13]; 3) and the use of the free fat mass index of the general population, stratified for age and sex, as reference value [5,8,33]. In the present study, we used MMI cut-offs adopted to identify the primary sarcopenia [1], for two reasons: the comparison of MMI in patients vs. age- and sex-matched controls cannot be routinely used for the diagnosis of sarcopenia, and the free fat mass index is not specific for muscle mass. In addition, using the same methodologic approach including both MMI and muscle strength, we compared, for the first time, the prevalence of sarcopenia in RA, AS and PsA. 

We found a similar prevalence of sarcopenia (about 20%) in all three rheumatic diseases, since the only two factors significantly influencing this condition, i.e. age and disability, were similar in RA, AS and PsA (Table 1). These findings apparently differ from the data reported in a recent study of Asian patients with RA, showing a higher prevalence of sarcopenia (37.1%) using the same methodologic approach [13]. However, the patients enrolled in this study were older compared to our patients (median age 65 (54.3–72.0) years vs. 56 (48.0–62.2)), and 1/3 were malnourished (we excluded all subjects with malnutrition, since this condition is recognized as one of the main causes of sarcopenia [34,35]). Another recent study of North African patients with AS [11] showed a higher prevalence of sarcopenia (34.3%), using a different methodologic approach (DXA and handgrip strength or physical performance test) and including an undetermined number of malnourished patients. The only study evaluating the prevalence of sarcopenia in patients with PsA estimated it to be about 13.7%. This study was conducted presumably in Caucasian patients and used the appendicular lean body mass index instead of MMI for the assessment of sarcopenia [12]. 

We observed a prevalence (10.5%) of presarcopenia in RA. This finding was similar to that reported by a Torii et al. (11.9%) using our methodologic approach [13]. On the other hand, we found a prevalence of 36.3% in AS, while a previous study found a higher value (50.4%) [11] using another methodologic approach. To our knowledge, the present study is the first to assess the prevalence of presarcopenia in PsA, which was 25.7%. On the basis of our finding, presarcopenia in PsA and AS patients was 2.5 and 3.6 times higher compared to RA, probably because males are more exposed to the risk of presarcopenia [36], and in our cohort RA patients were almost exclusively females, while the percentage of males significantly increased in PsA and even more in AS (Table 1). 

The secondary endpoint of our study was the evaluation of putative risk factors associated with sarcopenia. Although patients with the different rheumatic diseases were significantly different for sex, physical activity and inflammatory status (Table 1), both at univariate and multivariate analysis, the type of disease did not influence the risk of sarcopenia. When we analyzed other possible risk factors, at multivariate analysis, only age ≥60 years and the presence of a disability significantly influenced sarcopenia, while the CPR almost reached statistical significance. To further evaluate the role of inflammation in the pathogenesis of sarcopenia, in addition to previous analyses, we evaluated the multiple disease activity scores specific for each rheumatologic disease. DAS 28, CDAI, SDAI, DAPSA, ASDAS-CRP and ASDAS-ESR were not associated with sarcopenia. These findings were in agreement with those previously reported by others using DAS-28 in RA patients [13]. The only disease activity index that we found associated with sarcopenia was the BASDAI, which is the only score exclusively based on subjective patients’ evaluations. This association, which we observed in both genders, was in agreement with previous data reported in men [11]. 

A final comment concerns our finding on the lack of a protective effect of biologic therapy against sarcopenia, in contrast with the data reported in AR patients [13]. One possible explanation comes from the inclusion of all patients with rheumatic diseases in our analysis, comprising patients with AS, in whom sarcopenia is not influenced by biologic therapy [11]. However, the fact remains that our patients undergoing biologic therapy showed a higher prevalence of disability, which is per se a significant risk factor for sarcopenia. 

Our study suffers some limitations: (1) the data on the prevalence of sarcopenia were from a single centre; however, our patients were from a vast area of Southern Italy, and their number was comparable to those reported in other studies [4,5,7,8]; (2) we did not have a sex- and age-matched control population; however, to establish reliable reference cut-offs stratified by gender and age, it is necessary to perform large population studies, an aspect that goes beyond the aim of the present study; (3) the evaluation of inflammatory activity was based on disease scores and biochemical indexes of inflammation and not on the assessment of pro-inflammatory cytokines; (4) our results cannot apply to obese patients. 

## 5. Conclusions

We compared, for the first time, the prevalence of sarcopenia in male and female patients affected by RA, PsA and AS, demonstrating that its value is similar in the three diseases. On the other hand, presarcopenia was shown to significantly increase in PsA and AS compared to RA. Our analysis suggests that the only parameters associated with the development of sarcopenia are age and disability, while disease activity seems to have a lower influence on the risk of sarcopenia.

## Figures and Tables

**Table 1:** Demographic, clinical and therapeutic data in patients with the different rheumatic diseases analysed.

	Rheumatoid Arthritis	Psoriatic Arthritis	Ankylosing Spondylitis	*p*
N. of pts	76	70	22	
M/F	10/66	32/38	14/8	<0.001 *
Age	56.5 ± 8.8	55.3 ± 9.1	51.6 ± 8.8	0.08 ^†^
Body mass index (BMI) (kg/m^2^)	24.9 ± 3.2	25.6 ± 3.0	24.9 ± 2.5	0.37 ^†^
Caloric intake (Kcal/Kg/die)	24.8 ± 6.7	23.7 ± 6.8	24.8 ± 6.5	0.54 ^†^
Protein intake (g/Kg/die)	0.9 ± 0.2	0.8 ± 0.2	0.9 ± 0.2	0.39 ^†^
Physical activity level	1.3 ± 0.1	1.4 ± 0.2	1.4 ± 0.2	0.02 ^†^
Disease duration (years)	10.8 ± 8.2	11.1 ± 8.1	14.5 ± 8.4	0.16 ^†^
C-reactive protein (CRP) elevated (%)	39.7	7.4	5.2	<0.001 *
Erythrocyte sedimentation rate (ESR) elevated (%)	51.3	26.0	18.1	0.001 *
Biologic therapy (%)	65.7	78.5	86.3	0.07 *
Disability (%)	52.7	54.2	54.5	0.97 *

Unless specified, the data reported in the table are expressed as means ± SD. * By chi square; † By ANOVA.

**Table 2:** Variables associated with sarcopenia.

Variable	N. of Patients with Sarcopenia/Tot. (%)	Univariate		Multivariate	
		OR (95% CI)	*p*	OR (95% CI)	*p*
Rheumatoid arthritis (RA)	16/76 (21.0)	ref.		ref.	
Psoriatic arthritis (PsA)	14/70 (20.0)	0.9 (0.4–2.0)	0.87	1.1 (0.4–2.7)	0.79
Ankylosing spondylitis (AS)	5/22 (22.7)	1.1 (0.3–3.4)	0.86	1.8 (0.5–6.7)	0.33
Gender					
Female	20/112 (17.8)	ref.			
Male	15/56 (26.7)	1.6 (0.7–3.6)	0.18		
Age (years)					
<60	13/109 (11.9)	ref.		ref.	
≥60	22/59 (37.2)	4.3 (2.0–9.6)	<0.001	3.3 (1.4–7.7)	0.005
Calorie intake ^a^	-	1.0 (0.9–1.0)	0.19		
Protein intake ^b^	-	2.1 (0.5–8.9)	0.28		
Physical activity level (PAL) ^c^	-	0.2 (0.02–3.5)	0.32		
Disease duration ^d^	-	1.0 (0.9–1.0)	0.37		
C-reactive protein (CRP) *					
normal	26/137 (18.9)	ref.		ref.	
elevated	9/21 (42.8)	3.2 (1.2–8.3)	0.01	2.6 (0.8–7.8)	0.07
Erythrocyte sedimentation rate (ESR) **					
normal	18/105 (17.1)	ref.			
elevated	16/60 (26.6)	1.7 (0.8–3.7)	0.14		
Biologic therapy					
no	6/44 (13.6)	ref.			
yes	29/124 (23.3)	1.9 (0.7–5.0)	0.17		
Disability					
no	7/77 (9.0)	ref.		ref.	
yes	28/89 (31.4)	4.5 (1.8–11.2)	0.001	3.0 (1.2–7.9)	0.01

OR = odd ratio; ref. = reference value. ^a^ 25.7 ± 7.3 vs. 24.0 ± 6.6 kcal/kg in sarcopenic and non-sarcopenic patients, respectively; ^b^ 1.0 ± 0.3 vs. 0.9 ± 0.3 g/kg in sarcopenic and non-sarcopenic patients, respectively; ^c^ 1.3 ± 0.1 vs. 1.3 ± 0.2 in sarcopenic and non-sarcopenic patients, respectively; ^d^ 13.4 ± 9.8 vs. 10.9 ± 7.7 years in sarcopenic and non-sarcopenic patients, respectively; * CRP in the 9 patients with sarcopenia was 2.6 ± 2.5 times higher compared to upper normal limit (see also Materials and Methods); ** Elevated ESR in the 16 patients with sarcopenia was 2.8 ± 1.1 times higher compared to upper normal limit (see also Materials and Methods).
